# Disruption of chlorophyll metabolism and photosynthetic efficiency in winter jujube (*Ziziphus jujuba*) Induced by *Apolygus lucorum* infestation

**DOI:** 10.3389/fpls.2025.1536534

**Published:** 2025-03-11

**Authors:** Wei Tan, Qi Yin, Haipeng Zhao, Mengyao Wang, Xia Sun, Hui Cao, Deya Wang, Qingliang Li

**Affiliations:** ^1^ College of Food Science and Pharmaceutical Engineering, Zaozhuang University, Zaozhuang, China; ^2^ College of Life Sciences, Zaozhuang University, Zaozhuang, China; ^3^ College of Plant Protection, Shandong Agricultural University, Taian, China

**Keywords:** *Ziziphus jujuba*, *Apolygus lucorum*, chlorophyll degradation, chlorophyll synthesis, photosynthetic performance

## Abstract

*Apolygus lucorum*, a cosmopolitan Hemiptera insect, poses a significant threat to *Ziziphus jujuba* (jujube), causing symptoms such as mottled chlorosis. However, the mechanisms through which *A. lucorum* affects chlorophyll biosynthesis and degradation remain unclear. This study investigates the effects of *A. lucorum* infestation on chlorophyll metabolism and photosynthetic performance in winter jujube leaves. Results revealed a substantial reduction in chlorophyll a and b content, which was strongly correlated with decreases in key photosynthetic parameters, such as the Photochemical Performance Index (PI_ABS_) and electron transport efficiency (ψ(Eo)). Infestation downregulated genes critical for chlorophyll biosynthesis, such as *HEMA*, *HEMB*, and *chlG*, leading to a bottleneck in chlorophyll production. Concurrently, chlorophyll degradation pathways were upregulated, with genes like *PAO* and *RCCR* driving increased chlorophyll catabolism. This imbalance between reduced synthesis and accelerated degradation exacerbated chlorophyll loss, impairing photosynthetic capacity. Furthermore, the application of exogenous H_2_O_2_ intensified chlorophyll degradation, particularly in *A. lucorum*-infested leaves. The accelerated degradation of chlorophyll, coupled with reduced light-harvesting efficiency, contributed to oxidative stress and further impaired the photosynthetic machinery, despite an increase in antioxidant enzyme activity. These findings underline the critical role of chlorophyll metabolism in maintaining photosynthetic efficiency in winter jujube under *A. lucorum* infestation. It underscores the need for targeted strategies to protect chlorophyll synthesis and limit its degradation in order to mitigate the detrimental effects of *A. lucorum*-induced stress.

## 1 Introduction

The jujube tree, *Ziziphus jujuba*, primarily found in various regions of China, is known for its dual medicinal and ecological benefits (Cheng et al., 2020). Its nutritional components, like saponins and flavonoids, contribute to its antioxidant and anti-inflammatory effects, aiding in the prevention of chronic illnesses and enhancing health (Chen et al., 2017). Additionally, the polysaccharides in jujube fruits are recognized for their potential in combating cancer, particularly by hampering tumor growth and promoting the death of cancer cells (Sun et al., 2013; Ji et al., 2020; Corso et al., 2021). Remarkably adaptable, jujube thrives in harsh continental climates, enduring conditions like drought and poor soil, making it an ideal species for reforesting barren areas (Liu, 2006; Liu et al., 2020b). Jujube trees frequently suffer from a range of pests, including Hemiptera (true bugs), lepidopteran larvae such as *Ancylis sativa and Carposina sasakii*, and arachnids like spider mites and so on (Pan et al., 2021). In particular, the damage caused by *Apolygus lucorum* has gradually increased in recent years (Li et al., 2023). This increase in damage is linked to significant changes in agricultural practices, particularly the widespread application of genetically modified cotton ( (Lu et al., 2010).

*Apolygus lucorum* (Hemiptera: Miridae), an incomplete metamorphosis insect, has emerged as a significant pest in Asian jujube plantations (Lu et al., 2010; Pan et al., 2015). In China, the damage to jujube leaves caused by this pest increased dramatically from 20%-30% in 1997 to 80%-90% recently, posing a substantial threat to the industry (Li et al., 2023). The pest’s stylet mouthparts inflict severe harm to new leaves, adversely affecting the health and yield of the jujube trees (Lu et al., 2010). Additionally, infestation by *A. lucorum* leads to leaf mottling and chlorosis in jujube plants. It was generally believed that the yellowing of plant leaves is caused by a reduction in chlorophyll content.

Chlorophyll, the primary pigment responsible for photosynthesis, plays a vital role in light absorption, energy transfer, and photochemical reactions (Glazer, 1989; Stirbet et al., 2020). A positive correlation exists between chlorophyll content and photosynthetic capacity, making chlorophyll essential for plant growth and development (Sairam et al., 2002). Previous studies have demonstrated that the level of chlorophyll in plants was contingent upon the dynamic equilibrium between chlorophyll synthesis and degradation processes (vom Dorp et al., 2015; Szafranska et al., 2017). The biosynthesis of chlorophyll involves a complex, multistep pathway, with key intermediates such as glutamate (Glu), 5-aminolevulinic acid (ALA), porphobilinogen (PBG), and protoporphyrin IX (ProtoIX), leading to the formation of chlorophyll a and b (Bollivar, 2006; Wang and Grimm, 2015; Lin and Charng, 2021). The process of chlorophyll synthesis encompasses 16 steps, including ALA synthesis, ProtoIX synthesis, conversion from ProtoIX to Pchl, and subsequent conversion from Pchl to chlorophyll *a* and *b* (Bollivar, 2006; Wang and Grimm, 2015; Lin and Charng, 2021). This process is regulated by more than 20 genes, and disruptions at any stage can severely reduce chlorophyll production (Farouk and Al-Ghamdi, 2021). Various biological and abiotic stresses such as diseases, salt stress, and heat stress can disturb the delicate balance between chlorophyll synthesis and degradation processes, consequently causing alterations in chlorophyll levels (Tan et al., 2011; Jin et al., 2019; Štambuk et al., 2021).

Numerous studies have consistently demonstrated a decline in leaf chlorophyll content following infestation by various piercing-sucking pests (Gutsche et al., 2009; Touhidul Islam and Shunxiang, 2009; Li et al., 2013; Sytykiewicz et al., 2013; Huang et al., 2014; Golan et al., 2015; Li et al., 2024). For instance, the chlorophyll content in *Sorghum bicolor* leaves exhibited a significantly decrease subsequent to damage inflicted by the sugarcane aphid, *Melanaphis sacchari* (Carey et al., 2022). Similarly, wheat leaves displayed a marked reduction in chlorophyll content when subjected to attacks by grain aphids such as *Sitobion avenae*, *Schizaphis graminum*, and *Rhopalosiphum padi* (Liu et al., 2020a). Damage to cereals by *Schizaphis graminum* resulted in a loss of chlorophyllin and a concomitant reduction chlorophyll levels (Zhang et al., 2020). Host plants, including *Cucurbita pepo* and *Nicotiana tabacum*, experienced declines in both chlorophyll content and photosynthetic rates due to infestations by *Bemisia tabaci* (MEAM1) (Jimenez et al., 1995; McAuslane et al., 2004; Li et al., 2013). Futhermore, a range of other hosts such as *Vigna radiate*, *Vitis vinifera*, and *Gossypium* spp exhibited significant reductions in their chlorophyll contents following *A. lucorum* infestation (Luo et al., 2011; Li et al., 2016; Gao et al., 2022). Despite these findings, the precise mechanisms by which herbivorous insects reduce chlorophyll content in plants remain poorly understood.

Approximately 60~80% of jujube fruit yield is attributable to leaf-based photosynthesis. However, the interrelationship between chlorophyll content and photosynthetic performance in host plants subjected to *A. lucorum* infestation remained to be elucidated. Additionally, the impact of *A. lucorum* on the dynamics of chlorophyll synthesis and degradation in host leaves post-infestation was not yet fully understood. So, we investigated the variations in chlorophyll content of jujube infested by *A. lucorum*. We also examined precursor molecules involved in chlorophyll biosynthesis, chlorophyll degradation products, and the expression profiles of key genes implicated in both chlorophyll synthesis and degradation. This investigation focused on winter jujube leaves infested by *A. lucorum*. Winter jujube was chosen for the study due to its widespread cultivation in China and its observed susceptibility to *Apolygus lucorum*, as confirmed by preliminary field surveys, which showed a higher infestation rate compared to other jujube varieties. The overarching objective was to elucidate the mechanistic pathways through which *A. lucorum* influenced chlorophyll biosynthesis and degradation within the leaves of jujube.

## 2 Materials and methods

### 2.1 Plant and insect materials

The cultivation conditions for annual seedlings of winter jujube (*Ziziphus jujuba* cv. Dongzao) were consistent with those detailed in our previous study (Li et al., 2023). These seedlings were maintained in a climate-controlled chamber at a temperature of 28 ± 3°C, a relative humidity of 75 ± 5%, and a photoperiod of 16:8 h (Light: Dark) (Li et al., 2023).

For sourcing and rearing *A. lucorum* nymphs, we adhered to the protocols established by Li et al. (2023). Only newly emerged adult *A. lucorum* were selected for our experiments.

### 2.2 Plant infested by *A. lucorum*

Seedlings that had been germinated for one month from one-year-old jujube plants were used in the experiments. Each plant was exposed to twenty 3-day-old adult *A. lucorum*, enclosed in a 120-mesh nylon cage (60 cm × 60 cm × 100 cm). The procedure started at 18:00 hours. Control plants without *A. lucorum* were kept under the same conditions. After seven days, the insects were removed. The leaves were then immediately flash-frozen in liquid nitrogen and preserved at -80°C for further analysis, as per the methodology in Li et al. (2023).

### 2.3 Measurement of photosynthesis

Net photosynthetic gas exchange measurements were performed on leaves infested with *A. lucorum* in both treated and control groups of winter jujube. These measurements were conducted 7 days post-infestation using an open gas exchange system (Ciras-3, PP Systems, Hitchin, UK). Each set of conditions was replicated six times for statistical robustness.

### 2.4 Measurement of chlorophyll fluorescence transients

The methodology for measuring polyphasic chlorophyll fluorescence transients (OJIP) has been previously described (Li et al., 2013). In this study, OJIP transients were assessed in leaves infested with *A. lucorum* for both treated and control groups of winter jujube, 7 days following infestation. Measurements were carried out using a Plant Efficiency Analyzer (PEA; Hansatech Instruments Ltd., King’s Lynn and Norfolk PE32 1JL, UK), in accordance with the protocols established by Strasser and Srivastava, (1995).

The fluorescence transients were induced using red light at an intensity of approximately 3000 µmol m^-2^ s^-1^, provided by an array of six light-emitting diodes (peak wavelength 650 nm). Fluorescence signals were recorded over a time span ranging from 10 µs to 1 s. The data acquisition rates were set at 105 points per second for the initial 2 ms and 103 points per second thereafter. Each experimental condition, focusing on leaves at specific positions, was replicated six times for consistency.

### 2.5 Determination of pigments content

After a 7-day period, the contents of chlorophyll *a*, chlorophyll *b*, and carotenoids in leaves infested with *A. lucorum* were determined for both the treated and control groups of winter jujube. Leaf samples were collected from jujube trees and immediately placed in liquid nitrogen to preserve the integrity of the pigments. The samples were then stored at -80°C until further processing. Leaf samples were homogenized in 80% acetone. The chlorophyll content was measured using a Unicam UV 550 double-beam spectrophotometer (Thermo Spectronic, Cambridge, UK). The absorbance of the solution was recorded at 663 nm (for chlorophyll a), 645 nm (for chlorophyll b) and 470nm (for carotenoid), according to the protocol established by Lichtenthaler (1987). Each experimental condition was replicated six times.

### 2.6 Determination of precursors and degradation products of chlorophyll

Mg-ProtoIX, ProtoIX, and Pchl levels in *A. lucorum*-infested jujube leaves were assessed for treated and control groups using Hodgins and Van Huystee (1986) method. UroIII was quantified following Bogorad (1962), ALA per Morton (1975), and PBG using Bogorad (1962) again. Chlorophyllide analysis was done as described by Porra et al. (1989), Pheophorbide following Vergara-Domínguez et al. (2016), and Pheophytin per Edelenbos et al. (2001).

### 2.7 Quantitative real-time PCR for validation of the transcript levels

Jujube plant leaves were collected, immediately frozen at -80°C, and processed for total RNA extraction and qRT-PCR. A mix of leaves from the same treatment was used for three replicates. Using 100 mg of leaf tissue per sample, RNA was extracted in TRIzol, followed by first-strand synthesis with RevertAid Premium Reverse Transcriptase. qPCR was conducted on an ABI Stepone plus system with SG Fast qPCR Master Mix. Key genes in chlorophyll biosynthesis and degradation pathways were quantified relative to *ZjH3* using the 2-ΔΔCT method. Primers, listed in Supplementary Table S4, were designed based on GenBank’s *Z. jujuba* genome models.

### 2.8 ROS assay

Superoxide Anion (O_2_^-^) Measurement: The production of superoxide anion (O_2_^-^) in jujube leaves was quantified using the nitroblue tetrazolium (NBT) staining method as described by Zhang et al. (2015). Leaf samples (0.2 g) were homogenized in 5 mL of phosphate buffer (50 mM, pH 7.8). The homogenate was centrifuged at 10,000 × g for 15 minutes at 4°C. The supernatant was then mixed with 1 mL of 0.1% NBT solution and incubated in the dark at room temperature for 1 hour. After the reaction, the absorbance of the reaction mixture was measured at 530 nm using a spectrophotometer, with the NBT reduction indicating the presence of superoxide radicals. Results were expressed as absorbance per gram of fresh weight.

Hydrogen Peroxide (H_2_O_2_) Measurement: Hydrogen peroxide (H_2_O_2_) levels were determined using the potassium iodide (KI) method as described by Velikova et al. (2000). Leaf samples (0.2 g) were homogenized in 5 mL of 0.1% (w/v) trichloroacetic acid (TCA). The homogenate was centrifuged at 12,000 × g for 15 minutes at 4°C, and 0.5 mL of the supernatant was mixed with 0.5 mL of potassium phosphate buffer (pH 7.0, 10 mM) and 1 mL of 1 M KI solution. The reaction mixture was incubated in the dark for 1 hour at room temperature. The absorbance was measured at 390 nm using a spectrophotometer, and H_2_O_2_ content was calculated based on a standard curve of known H_2_O_2_ concentrations. The results were expressed as µmol per gram of fresh weight.

### 2.9 Antioxidant enzyme assay

For antioxidant enzyme activity analysis, 0.2 g of leaf tissues was homogenized in 1.6 mL of 50 mM phosphate buffer, then centrifuged at 12,000 g, 4°C for 20 min. The supernatant was used for assays. SOD activity was measured following Giannopolitis and Ries (1977), POD activity as per Tao et al. (2020), CAT activity according to Dhindsa et al. (1982), and APX activity using Nakano and Asada (1981) method.

### 2.10 Plants treated with hydrogen peroxide

To investigate the effects of H_2_O_2_ on the chlorophyll metabolism in jujube leaves during *A. lucorum* infestation, we applied exogenous H_2_O_2_ through foliar spraying. Three days subsequent to the introduction of *A. lucorum*, jujube plants were subjected to a treatment regimen involving the application of H_2_O_2_ solutions at concentrations of 50 mM and 100 mM. This treatment was administered daily to the entirety of each plant. Control groups were treated with an equivalent volume of water. Each experimental and control treatment was replicated six times. Upon reaching the seventh day of exposure to *A. lucorum*, the insects were carefully removed. Subsequently, foliar samples were promptly flash-frozen in liquid nitrogen and preserved at a temperature of -80°C for future analytical procedures.

### 2.11 Statistical analysis

A t-test was performed to assess whether differences in the results of independent experiments were statistically significant using SPSS version 13.0 software (SPSS, Chicago, IL, USA), and comparisons between the mean values were made by the least significant difference (LSD) at a 0.05 probability level. The correlation analysis was performed using the Pearson correlation method.

## 3 Results

### 3.1 Photosynthetic pigment content in jujube leaves after *A. lucorum* infestation

**Figure 1:**
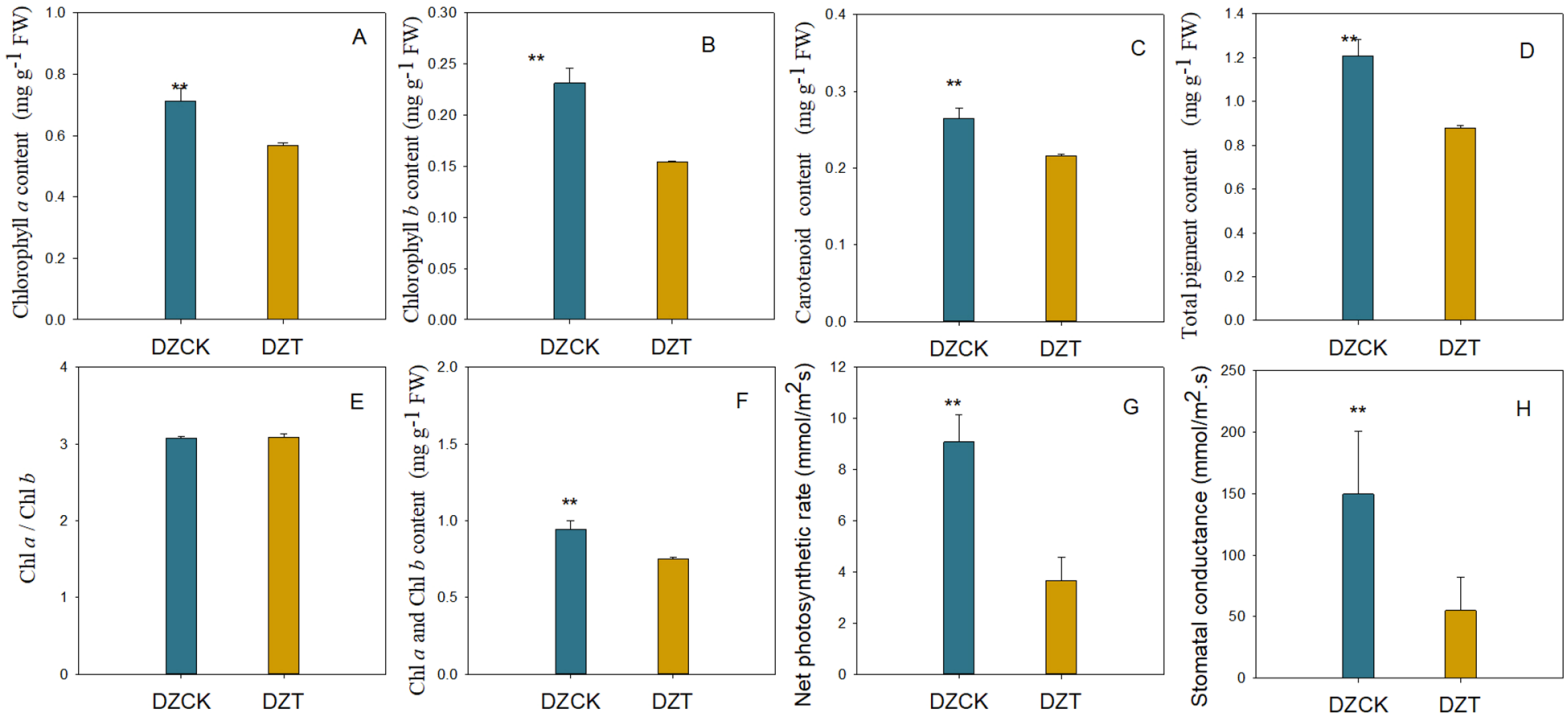
Photosynthetic pigments content and gas exchange parameters in the leaves of different jujube varieties infested by *A. lucorum.* **(A)** Content of Chlorophyll *a* in different jujube leaves. **(B)** Content of Chlorophyll *a* in different jujube leaves. **(C)** Content of carotenoid in different jujube leaves. **(D)** Total pigment content in different jujube leaves. **(E)** Chla/Chlb in different jujube leaves. **(F)** Content of Chla and Chlb in different jujube leaves. **(G)** Net photosynthetic rate in different jujube leaves. **(H)** Stomatal conductance in different jujube leaves.DZCK, winter jujube without *A. lucorum*; DZT, winter jujube infested by *A. lucorum*. Data (mean ± SE) were tested for significant differences using Student’s *t* test (* *p* < 0.05, ** *p* < 0.01).

Following seven days of infestation by *A. lucorum*, significant reductions were observed in the photosynthetic pigments of winter jujube leaves. Specifically, chlorophyll *a* content decreased by 34.25% (t = 6.231, p = 2.51×10^-4^) as shown in **Figure 1**, and chlorophyll *b* content decreased by 25.73% (t = 6.414, p = 2.06×10^-4^), detailed in **Figure 1**. The total chlorophyll content reflected this downward trend, showing a reduction of 24.79% (t = 6.305, p = 2.31×10^-4), as indicated in **Figure 1**. Additionally, the carotenoid levels also fell by 22.64% (t = 6.614, p = 1.67×10^-4^) (**Figure 1**). Despite these significant decreases in individual and total pigment contents, the chlorophyll a/b ratio (Ca/Cb) remained stable with no significant changes observed (**Figure 1**).

### 3.2 Photosynthetic efficiency and its correlation with pigment content

The net photosynthetic rate (Pn) of winter jujube leaves exhibited a pronounced decline, decreasing by 59.61% (t = 8.970, p = 2.10×10^-5^) (**Figure 1**). This substantial drop in photosynthetic activity underscores the severe impact of *A. lucorum* damage on the physiological functionality of jujube leaves.

In winter jujube leaves, significant positive correlations were observed between the decreases in chlorophyll *a* and *b* contents and the net photosynthetic rate (Pn). Specifically, the Pearson correlation coefficient for chlorophyll *a* was 0.847 with a p-value of 0.008, while for chlorophyll *b* it was 0.898 with a p-value of 0.002 (**Table 1**). Similarly, the overall decrease in total chlorophyll content also showed a positive correlation with the reduction in Pn, with a correlation coefficient of 0.864 (p = 0.04), as reported across **Table 1**. Conversely, the decrease in carotenoid content did not exhibit a significant correlation with changes in Pn (**Table 1**). These findings indicate that while the chlorophyll components are closely linked with the photosynthetic activity of the leaves, carotenoids may be influenced by different or additional factors that do not directly correlate with the net photosynthetic rate.

**Table 1:** Correlation analysis of chlorophyll and carotenoid content with net photosynthetic rate in winter jujube leaves infested by *apolygus lucorum*.

	Pearson correlation coefficient	*p*-value	Y=a+bx
Chlorophyll *a*	0.847	0.0080	Y=-12.329 + 29.655X
Chlorophyll *b*	0.898	0.002	Y=-13.271 + 95.409X
Carotenoid	0.308	0.458	Y=2.0451 + 20.025X
Chlorophyll *a*+ *b*	0.864	0.006	Y=-13.188 + 23.442X

This table displays the Pearson correlation coefficients, p-values and linear regression equations (Y=a+bx) describing the relationships between chlorophyll a, chlorophyll b, carotenoid content, and net photosynthetic rate (Pn) in winter jujube leaves under *Apolygus lucorum* infestation.

### 3.2 Differentially expressed genes enriched go terms related to photosynthesis and chlorophyll

We also highlighted differential transcriptomic expressions from RNA-seq in winter jujube leaves following *A. lucorum* infestation. Utilizing Gene Ontology (GO) analysis, we identified significant *A. lucorum* infestation alterations in pathways predominantly related to photosynthesis and chlorophyll metabolism.

**Figure 2:**
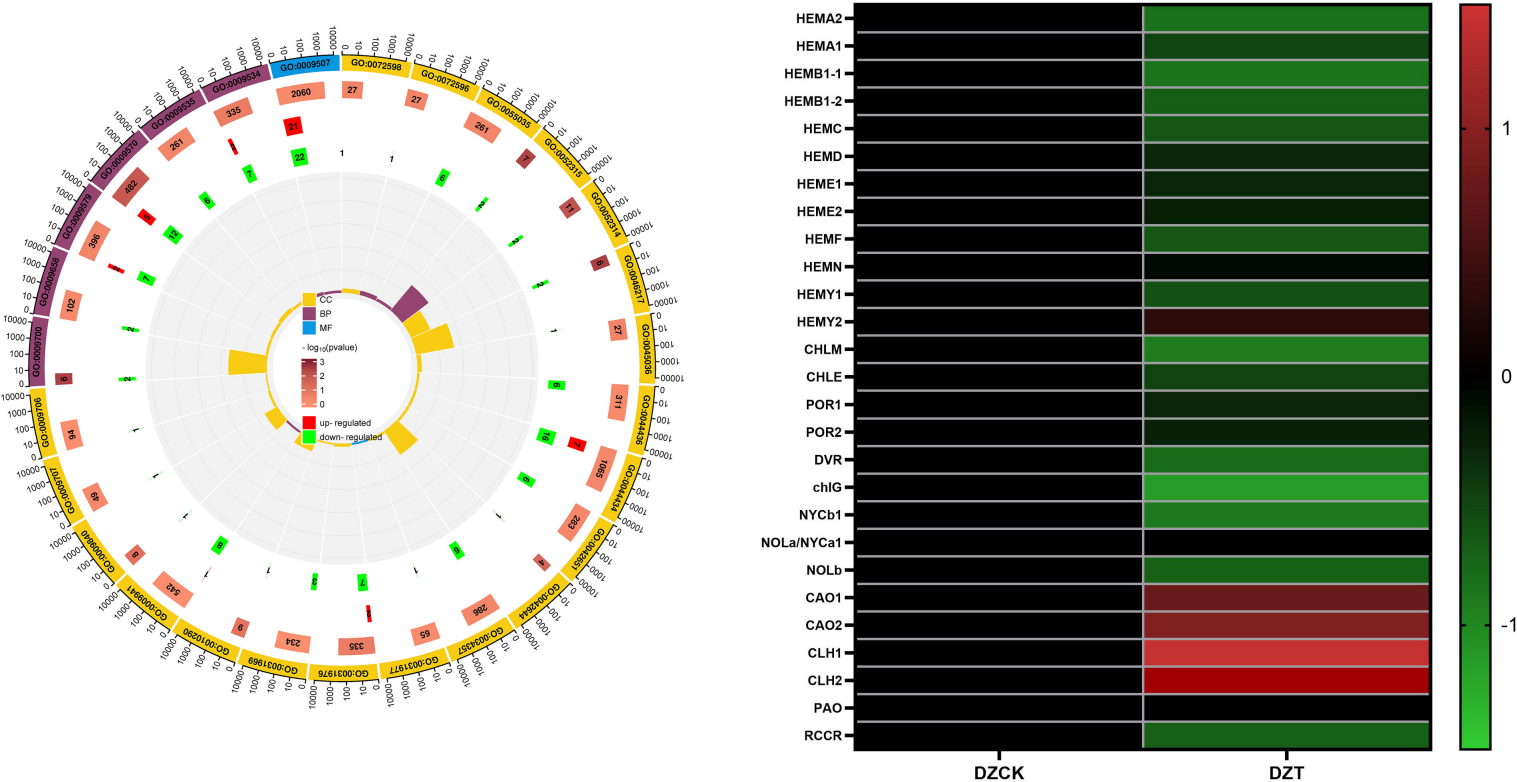
Transcriptome Analysis. **(A)** GO Terms in Winter Jujube. **(B)** Transcription level of genes related to chlorophyll metabolism in leaves of DZCK and DZT.

In winter jujube, 27 affected pathways were primarily localized within chloroplast and thylakoid components. Notable among these were the chloroplast pathway (GO: 0009507) with 43 differentially expressed genes (DEGs), chloroplast part (GO: 0044434) with 23 DEGs, and chloroplast stroma (GO: 0009570) with 17 DEGs (**Figure 2**). These changes suggest a substantial reprogramming of chloroplast functions, possibly impacting photosynthetic efficiency and stress adaptation mechanisms.

It was observed that *A. lucorum* infestation leads to notable transcriptomic changes, particularly in pathways related to chloroplast functions, which are essential for photosynthesis and chlorophyll metabolism. Following the infestation, gene expression in winter jujube leaves showed alterations in chlorophyll metabolism-related genes. Specifically, certain transcription factors (TFs) involved in chlorophyll biosynthesis exhibited a tendency to be downregulated, while others related to chlorophyll degradation showed a trend of upregulation (**Figure 2**).

### 3.3 qRT-PCR verification of the genes involved in chlorophyll metabolism

To validate the reliability of RNA-Seq data, twelve differentially expressed genes (DEGs) involved in chlorophyll metabolism were analyzed using qRT-PCR. Consistency between the qRT-PCR results and RNA-Seq data confirmed the reliability of the DEGs identified within the assembled transcriptome.

**Figure 3:**
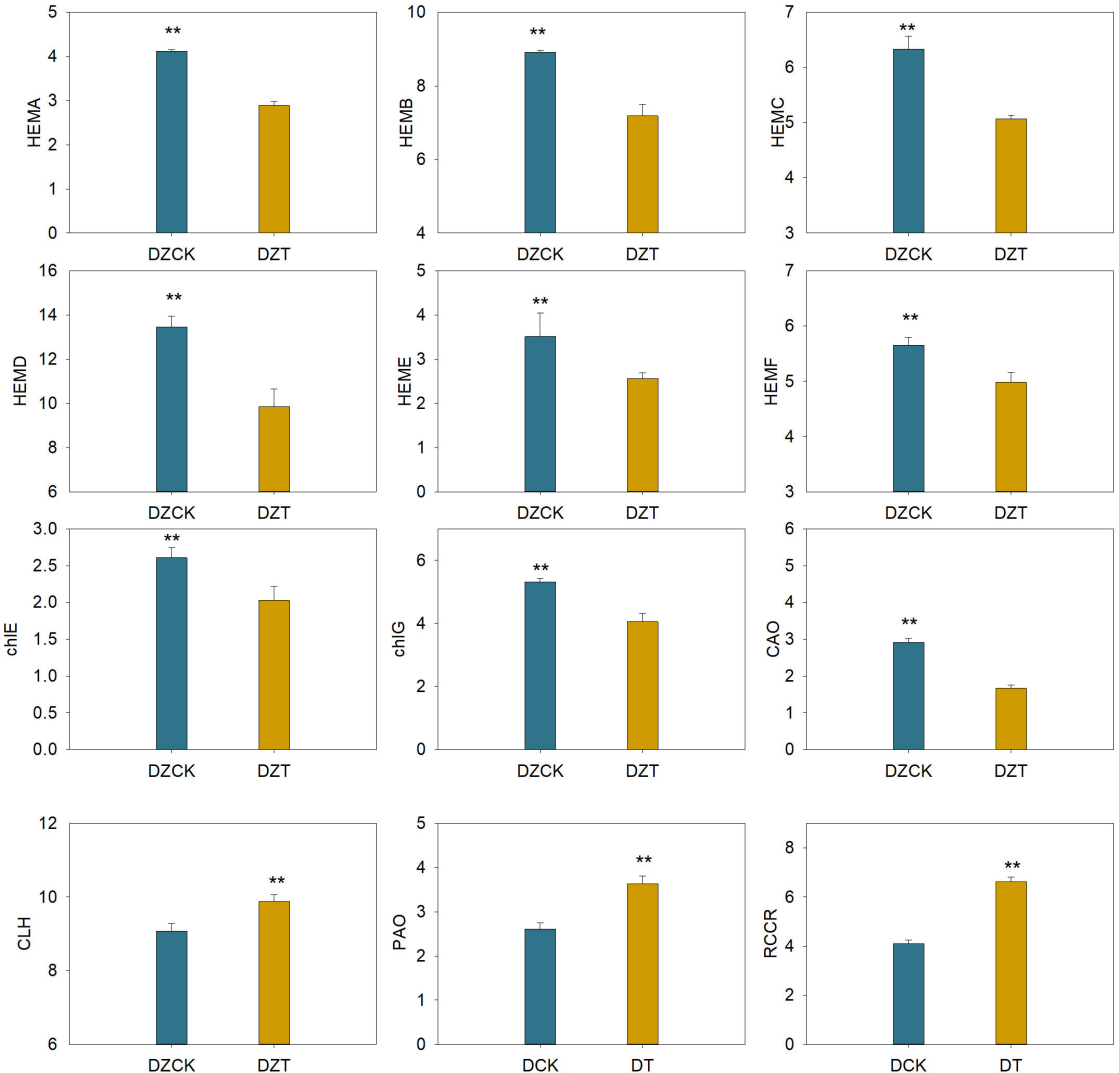
Effect of *Apolygus lucorum* infestation on the transcription level of genes related to chlorophyll metabolism in leaves of different jujube varieties. DZCK, winter jujube without *A. lucorum*; DZT, winter jujube infested by *A. lucorum*.

**Figure 4:**
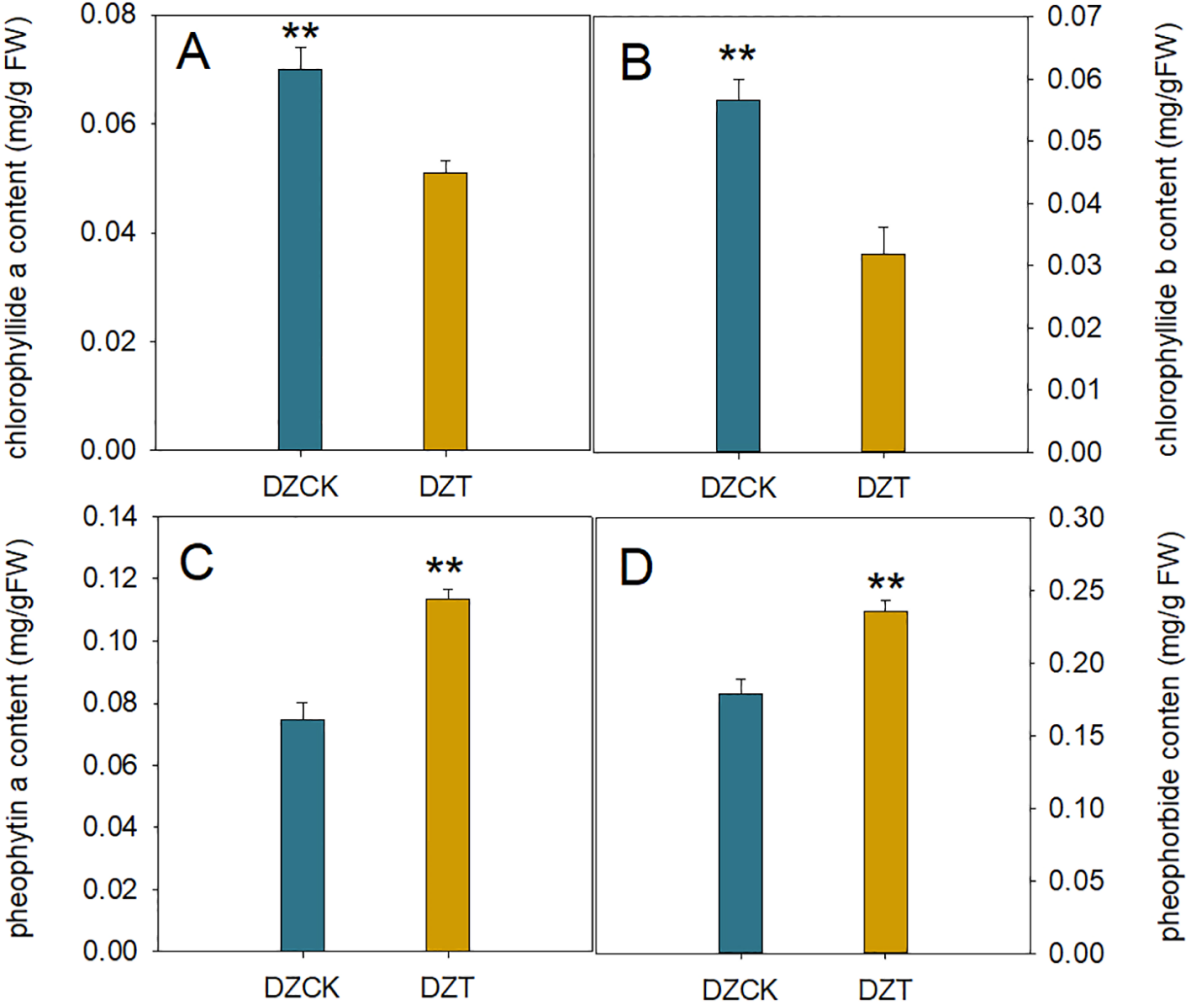
Impact of *Apolygus lucorum* infestation on Chlorophyll Degradation Products in *Ziziphus jujuba* Leaves. **(A)** Chloropryllide *a* content in different jujube leaves. **(B)** Chloropryllide *b* content in different jujube leaves. **(C)** Pheophytin *a* content in different jujube leaves. **(D)** heophorbide content in different jujube leaves. DZCK,winter jujube without *A. lucorum;* DZT, winter jujube infested by *A. lucorum.* Data (mean ± SE) were tested for significant differences using Student’s *t* test (* *p* < 0.05, ** *p* < 0.01).

Notably, the gene *HEMA* (Glutamyl-tRNA-reductase), responsible for the initial step in chlorophyll synthesis—converting glutamate to *ALA*—showed a significant decrease of 29.82% in expression in winter jujube post-infestation (P<0.05, **Figure 3**). Similarly, *HEMB* (Porphobilinogen Synthase), which catalyzes the conversion of ALA to PBG, was downregulated by 19.40% (**Figure 3**). Further down the pathway, *HEMC* (Hydroxymethylbilane Synthase) and *HEMD* (Uroporphyrinogen III Synthase), critical for the transformation of PBG into uroporphyrinogen III, showed decreased expressions by 19.95% and 26.75%, respectively (**Figure 4**). The subsequent conversion enzymes *HEME* (Coproporphyrinogen Synthase) and HEMF (Coproporphyrinogen III Oxidase) involved in producing protoporphyrin IX also exhibited declines of 26.94% and 11.92%, respectively (**Figure 3**). *chlG* (Chlorophyllide Synthase), essential for converting chlorophyllide to chlorophyll, showed a 23.69% decrease in expression following infestation (**Figure 3**). *CAO* (Chlorophyllide a Oxygenase), which plays a role in converting chlorophyllide a to b, also displayed a significant reduction of 42.93% in expression levels (**Figure 3**).

On the chlorophyll catabolic side, *CLH* (Chlorophyllase) exhibited a slight increase in expression by 5.41%, indicating enhanced degradation activity (**Figure 3**). The expression of *PAO* (Pheophorbide a Oxygenase), critical for chlorophyll catabolism, increased by 39.15%, and *RCCR* (Red Chlorophyll Catabolite Reductase), involved in further breaking down the chlorophyll molecule, showed an upsurge of 61.37% (**Figure 3**).

These data underline a significant shift in the chlorophyll metabolism pathway in winter jujube following *A. lucorum* infestation, marked by the downregulation of biosynthetic genes and upregulation of genes involved in chlorophyll degradation.

### 3.4 Impact of *A. lucorum* infestation on the levels of chlorophyll precursors in leaves of winter jujube

**Figure 5:**
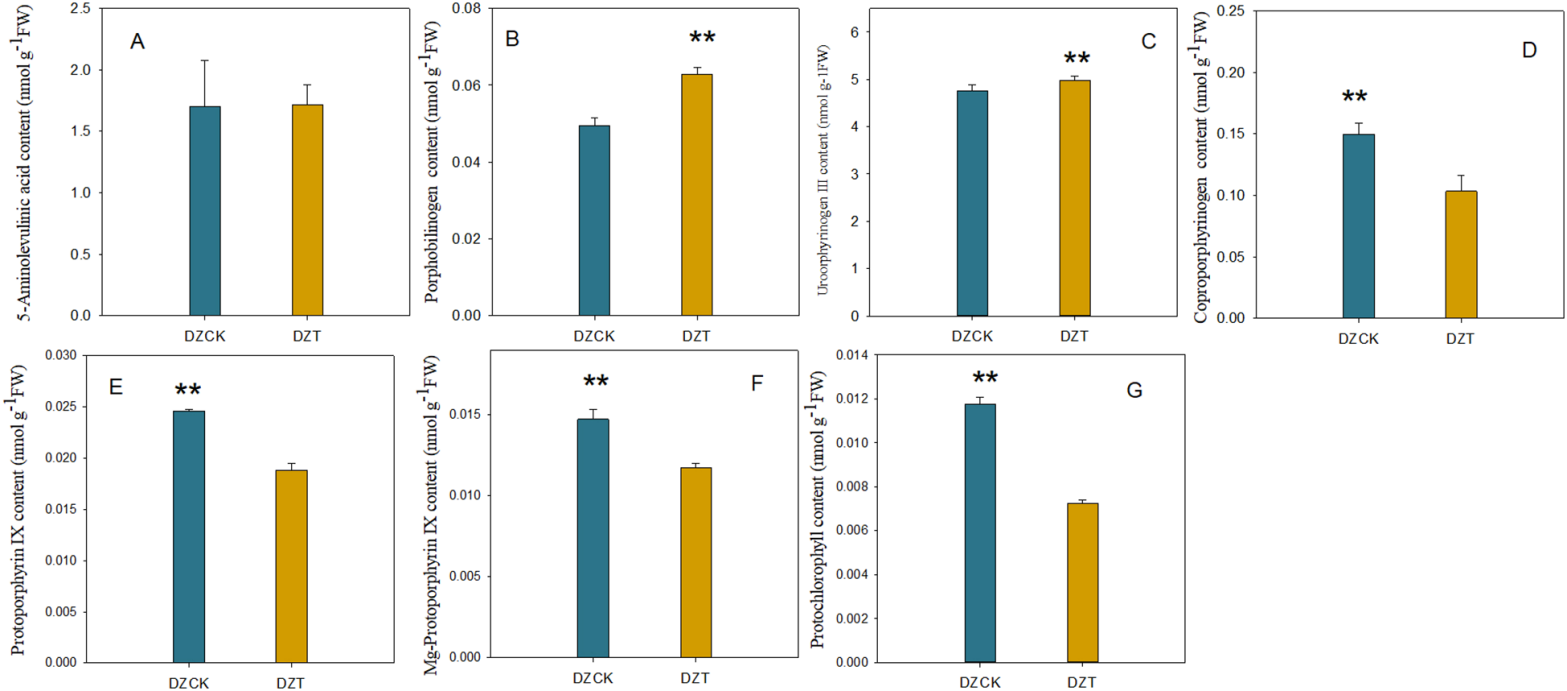
Impact of *Apolygus lucorum* infestation on the levels of chlorophyll precursors in leaves of different jujube cultivars. **(A)** ALA content in different jujube leaves. **(B)** PBG content in different jujube leaves. **(C)** UrogenIII content in different jujube leaves. **(D)** Coprogen content in different jujube leaves. **(E)** ProtoIX content in different jujube leaves. **(F)** Mg-ProtoIX content in different jujube leaves. **(G)** PchI content in different jujube leaves. DZCK, winter jujube without *A. lucorum;* DZT, winter jujube infested by *A. lucorum.* Data (mean ± SE) were tested for significant differences using Student’s *t* test (* *p* < 0.05, ** *p* < 0.01).

Exposure to *A. lucorum* led to varying responses in the biochemical composition of winter jujube leaves. Notably, the content of ALA did not significantly change compared to the control (t = -1.306, p = 0.271), indicating resilience in this metabolite’s levels despite infestation. In contrast, significant alterations were observed in several other porphyrin pathway intermediates. Specifically, the PBG content increased by 19.31% (t = -9.414, p = 0.003) as depicted in **Figure 5**, and UrogenIII content showed a moderate increase of 4.73% (t = -13.026, p = 2.04×10^-4^) illustrated in **Figure 5**. However, downstream metabolites experienced substantial reductions; Coprogen content decreased by 30.99% (t = 7.216, p = 0.003) and ProtoIX by 23.46% (t = 58.763, p < 0.001) (**Figure 5D**). Similar decreases were noted for Mg-ProtoIX and PchI, with reductions of 20.41% (t = 65.061, p < 0.001) and 38.33% (t = 263.610, p < 0.001), respectively.

### 3.5 Impact of *A. lucorum* infestation on the levels of chlorophyll degradation in leaves of winter jujube

After the infestation by *A. lucorum*, significant changes were observed in the chlorophyll metabolism within winter jujube leaves. Notably, the content of chlorophyllide *a*, an intermediate in chlorophyll synthesis and degradation pathways, decreased by 27.16% relative to the control (t = 9.900, p < 0.001) (**Figure 4**). Chlorophyllide b also showed a substantial decrease, dropping by 43.91% (t = 12.396, p < 0.001) (**Figure 4**).

Concurrently, there was a notable increase in the content of pheophytin *a*, the direct Mg-dechelation product of chlorophyll, which rose by 52.57% compared to the control (t = -14.716, p < 0.001) (**Figure 4**). Similarly, the content of pheophorbide, a further degradation product produced by the action of Mg-dechelatase, also increased by 51.06% (t = -9.199, p < 0.001) (**Figure 4**). This rise in degradation products correlates with changes in the Pn, with a significant negative correlation observed between *Pn* and pheophorbide content (Pearson correlation coefficient = -0.556, n = 24, p = 0.011), as depicted in **Figure 3**. These findings suggest that *A. lucorum* infestation significantly disrupts the chlorophyll metabolism, leading to decreased levels of chlorophyllide and an increase in its degradation products, which may contribute to the observed decline in photosynthetic efficiency in winter jujube leaves.

The infestation by *A. lucorum* markedly impacted the physiological status and environmental adaptability indicators in winter jujube leaves, as evidenced by substantial decreases in the ratios of chlorophyll and chlorophyllide to their respective degradation products. Specifically, the ratio of chlorophyll to pheophorbide decreased by 54.73% compared to the control (t = 69.367, p < 0.001), and the ratio of chlorophyllide to pheophorbide also fell by 51.55% (t = 52.441, p < 0.001) (**Table 2**). Furthermore, the ratio of chlorophyll to pheophytin showed an even greater decrease of 63.45% (t = 45.582, p < 0.001), and the ratio of chlorophyllide to pheophytin declined by 60.87% (t = 51.381, p < 0.001) (**Table 2**).

**Table 2:** Ratios of chlorophyll and chlorophyllide to pheophorbide and pheophytin in winter jujube leaves.

Treatments	chlorophyll/pheophorbide	chlorophyllide/pheophorbide	chlorophyll/pheophytin	chlorophyllide/pheophytin
**DZCK**	5.29 ± 0.077 c	0.71 ± 0.016 c	11.782 ± 0.156 c	1.582 ± 0.032 c
**DZT**	2.395 ± 0.019 d	0.344 ± 0.007 d	4.306 ± 0.35 d	0.619 ± 0.048 d

This table displays the ratios of chlorophyll to pheophorbide, chlorophyllide to pheophorbide, chlorophyll to pheophytin, and chlorophyllide to pheophytin in winter jujube (DZ) leaves under different treatments (CK: Control, T: Apolygus lucorum infested). The values represent means from 6 replications with standard deviations (± SD). Different letters (a, b, c, d) indicate significant differences among treatments based on statistical analysis.

These reductions in key ratios not only highlight a shift towards increased degradation over synthesis in the chlorophyll metabolism but also underscore a potential compromise in the photosynthetic efficiency and stress adaptability of winter jujube leaves under *A. lucorum* infestation.

### 3.6 Photosystem activity and chloroplast quantity in jujube leaves

The infestation of *A. lucorum* significantly affected the photosystem activity and chloroplast function in winter jujube leaves. Key indicators of photosystem performance demonstrated substantial reductions following infestation. The Photochemical Performance Index (PI_ABS_), an indicator of overall light energy conversion and electron transfer efficiency, showed a significant decrease of 60.71% (t = -4.251, p = 0.038) in winter jujube leaves compared to the control (**Table 3**). A marked reduction was observed in the maximum quantum efficiency of Photosystem II (Fv/Fm), which reflects the maximum efficiency of PSII. In winter jujube leaves, Fv/Fm decreased by 10.03% (t = 10.345, p = 0.001), indicating reduced photochemical efficiency under dark-adapted conditions (**Table 3**). The Fv/Fo ratio, which measures PSII potential activity, also decreased significantly. Fv/Fo was reduced by 35.03% (t = 9.671, p = 2.06 × 10^-4^) following *A. lucorum* infestation, further indicating a loss of photosynthetic potential in the chloroplasts (**Table 3**).

**Table 3:** Changes in chlorophyll fluorescence parameters in winter jujube leaves after infestation by *apolygus lucorum*.

Treatments	Fo/Fm	Fv/Fm	PI_ABS_	Fv/Fo	V_J_	VI	RC/Csm
**DZCK**	0.214 ± 0.009 c	0.786 ± 0.009 b	30.275 ± 8.321 a	3.683 ± 0.199 a	0.58 ± 0.043 b	0.678 ± 0.031b c	641.696 ± 29.048 b
DZT	0.295 ± 0.011 a	0.705 ± 0.012 d	11.895 ± 2.692 b	2.393 ± 0.132 c	0.762 ± 0.061 a	0.776 ± 0.028 a	303.057 ± 5.157 d

This table presents the changes in chlorophyll fluorescence parameters (Fo/Fm, Fv/Fm, PI_ABS_, Fv/Fo, VJ, VI, RC/Csm) in winter jujube (DZ) leaves following infestation by *Apolygus lucorum*, with control and treated (T) conditions. The values represent means with standard deviations (± SD), and they reflect alterations in chlorophyll fluorescence parameters, indicating physiological responses to infestation by the *Apolygus lucorum*.

The V_*j*_ parameter, representing the level of closure in PSII reaction centers, increased by 31.54% (t = -4.697, p = 0.016) in winter jujube leaves, suggesting an inhibition of electron transfer within the chloroplasts, particularly at the primary electron acceptor Q_A_ (**Table 3**). The V_*j*_ parameter, which reflects the relative fluorescence at the I-step and evaluates the activity of Photosystem I (PSI), also increased by 14.46% (t = -4.374, p = 0.008). This indicates impaired electron flow through PSI in chloroplasts subjected to *A. lucorum* infestation (**Table 3**).

A significant reduction was also found in the density of active reaction centers (RC/CS) per unit area. RC/CS in winter jujube leaves decreased by 52.77% (t = 13.658, p = 0.001), reflecting a decline in the number of functional reaction centers within the chloroplasts (**Table 3**).

### 3.7 Light energy utilization and electron transfer in photosynthetic structure of jujube leaves chloroplasts

**Figure 6:**
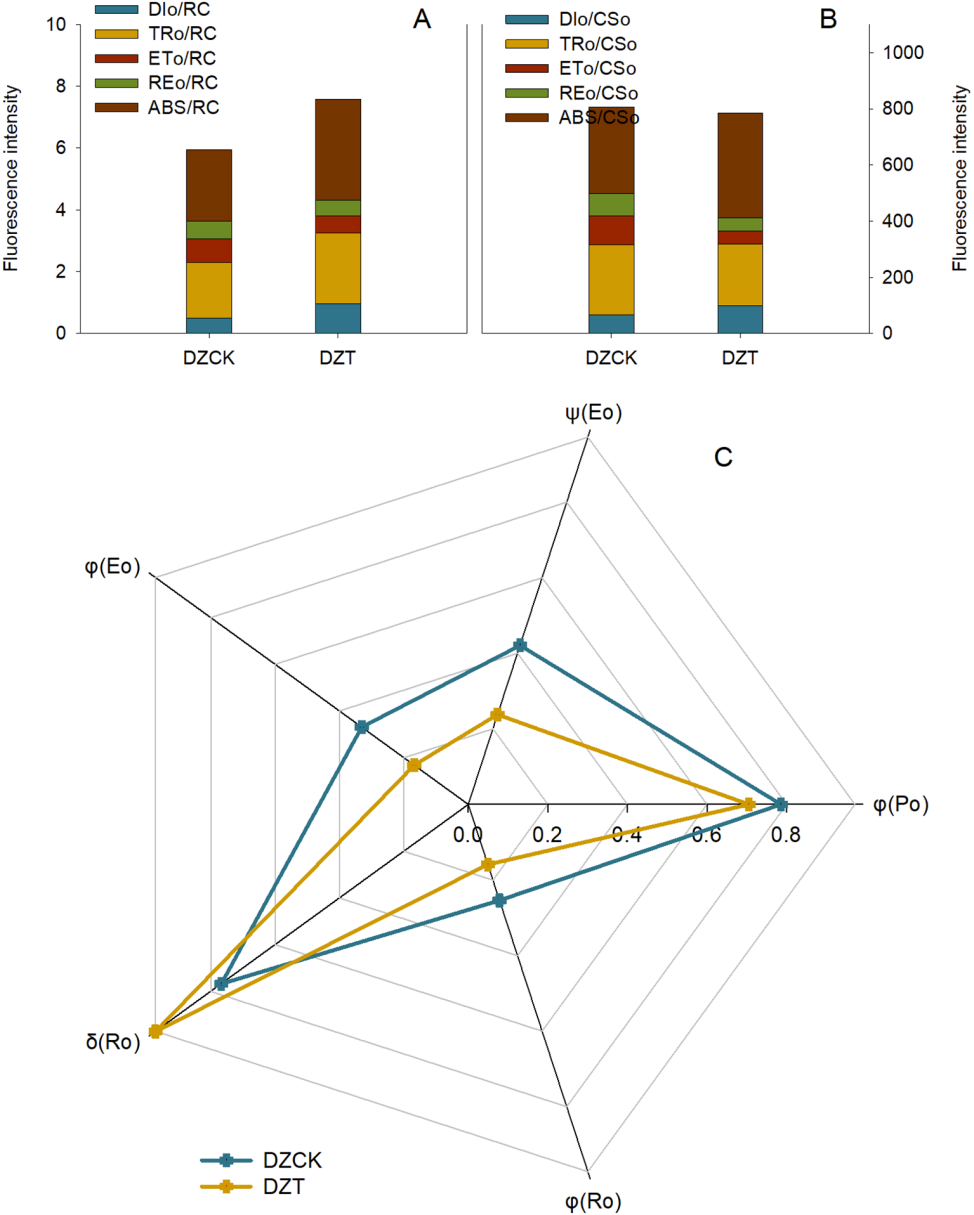
Evaluating the Effects of *Apolygus lucorum* Infestation on Photosynthetic Energy Dynamics in *Ziziphus jujuba* Leaves. **(A)** Energy metabolism per Reaction Center in jujube leaves; **(B)** Energy metabolism per cross sction in jujube leaves; **(C)** Electron transfer in jujube leaves. DZCK,winter jujube without *A. lucorum;* DZT,winter jujube infested by *A. lucorum*.

The impact of *A. lucorum* on the light energy utilization and electron transfer processes in winter jujube chloroplasts was quantified using multiple photochemical parameters. The Absorption Flux per Reaction Center (ABS/RC), which quantifies the number of photons absorbed by each reaction center, increased significantly by 41.49% (t = -4.551, p = 0.023) in winter jujube leaves relative to the control (**Figure 6**). Similarly, the Trapped Energy Flux per Reaction Center (TRo/RC), representing the amount of energy trapped by PSII reaction centers, increased by 26.98% (t = -3.749, p = 0.031) post-infestation (**Figure 6**). In contrast, the Electron Transport per Reaction Center (ETo/RC), which measures the efficiency of electron transport between Q_A_ and Q_B_ in PSII, decreased significantly by 28.96% (t = 3.716, p = 0.045) (**Figure 6**). This reduction reflects impaired electron flow within the photosystems following infestation.

Energy dissipation also increased significantly after infestation, as indicated by the Dissipation per Active Reaction Center (DIo/RC), which measures the amount of energy dissipated as heat. DIo/RC increased by 95.69% (t = -5.757, p = 0.017), highlighting reduced energy conversion efficiency and increased non-photochemical dissipation (**Figure 6**).

The Reduction of Electron Transport per Reaction Center (REo/RC), reflecting the reduction state of electron acceptors such as NADP^+^, decreased by 11.91% (t = 3.942, p = 0.013), suggesting impaired electron flow within the chloroplasts post-infestation (**Figure 6**).

Analysis of the Absorption Flux per Cross Section (ABS/CSo) revealed a significant increase of 22.04% (t = -3.104, p = 0.036) in winter jujube leaves (**Figure 6**), while Dissipation per Cross Section (DIo/CSo), which measures energy dissipation across the leaf surface, increased by 48.66% (t = -4.054, p = 0.027) (**Figure 6**). Furthermore, Electron Transport per Cross Section (ETo/CSo) decreased significantly by 56.097% (t = 3.716, p = 0.045), indicating a substantial decline in electron flow efficiency across the leaf surface post-infestation (**Figure 6**). Similarly, the Reduction of Electron Transport per Cross Section (REo/CSo), crucial for NADPH production, decreased by 42.68% (t = 3.942, p = 0.013) (**Figure 6**).

The Electron Transfer Efficiency (ψ(Eo)), which quantifies the efficiency of electron transfer between Q_A_ and Q_B_ in PSII, was reduced by 43.49% (t = 4.697, p = 0.016) after *A. lucorum* infestation (**Figure 6**). The Radiation Quantum Yield in Photoreactions (φ(Ro)), which evaluates the efficiency of absorbed photons used in photochemical reactions, also decreased by 37.58% (t = 5.136, p = 0.004) (**Figure 6**). The Photogenerated Electron Transfer Quantum Yield (φ(Eo)), which measures the efficiency of absorbed photons in facilitating electron transfer, decreased by 43.49% (t = 5.073, p = 0.008) (**Figure 6**). Lastly, the Quantum Yield of Non-Photochemical Consumption (δ(Ro)), which indicates the proportion of absorbed photons used in non-photochemical processes, increased by 37.58% (t = -2.393, p = 0.042), suggesting increased energy dissipation as heat (**Figure 6**).

### 3.8 ROS content and antioxidant enzyme activity in leaves of different jujube varieties

**Figure 7:**
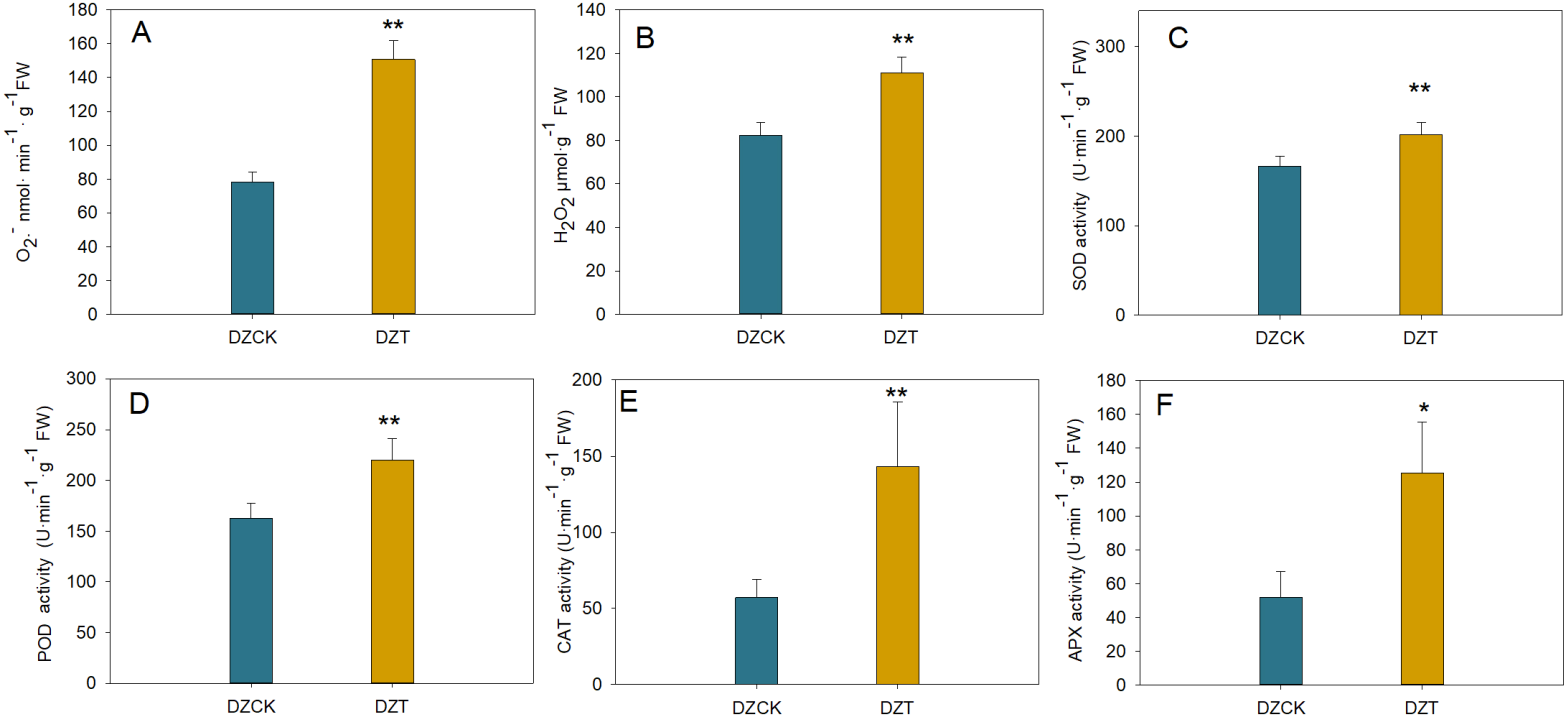
Quantitative Assessment of Reactive Oxygen Species and Antioxidant Enzymatic Responses in *Ziziphus jujuba* Leaves Subject to *Apolygus lucorum* Infestation. **(A)** Oxygen (O2) content across different jujube leaf samples. **(B)** Hydrogen peroxide (H2O2) levels in diverse jujube leaves. **(C)** Malondialdehyde (MDA) content indicating lipid peroxidation in various leaves. **(D)** Superoxide dismutase (SOD) activity across different leaf samples. **(E)** Peroxidase (POD) activity measurements in jujube leaves. **(F)** Catalase (CAT) activity levels in different leaf samples. **(G)** Ascorbate peroxidase (APX) activity in jujube leaves. **(H)** Polyphenol oxidase (PPO) activity across various leaf samples. Leaf samples are categorized as follows: DZCK,winter jujube without *A. lucorum;* DZT,winter jujube infested by *A. lucorum*. Significant differences were assessed using Student’s t-test (*p < 0.05, **p < 0.01*)*.

Following *A. lucorum* infestation, the superoxide anion (O_2_^-^) content in winter jujube leaves increased by 67.33% (t = -25.239, p = 2.6×10^-4^) compared to the control. Similarly, H_2_O_2_ levels, which can affect chloroplasts by causing oxidative damage, increased by 53.89% (t = -86.284, p < 0.01) (**Figure 7A**).

The malondialdehyde (MDA) content, an indicator of lipid peroxidation and potential membrane damage in chloroplasts, increased by 71.58% (t = -36.660, p < 0.001) in winter jujube leaves (**Figure 7**).

In response to this oxidative stress, antioxidant enzyme activities increased. The activity of superoxide dismutase (SOD), which mitigates oxidative damage in chloroplasts by converting O_2_^-^ into less toxic molecules, rose by 21.21% (t = -19.954, p = 0.001) (**Figure 7**). Similarly, the activities of other key antioxidant enzymes, ascorbate peroxidase (APX), peroxidase (POD), and catalase (CAT), which detoxify H_2_O_2_ within chloroplasts, increased by 1.42-fold (t = -5.417, p = 0.012), 34.92% (t = -7.068, p = 0.002), and 1.50-fold (t = -5.360, p = 0.006), respectively (**Figures 7E, F**).

### 3.9 Impact of exogenous H_2_O_2_ treatment on chlorophyll content and chlorophyll degradation ratios in winter jujube leaves post-infestation by *A. lucorum*

**Figure 8:**
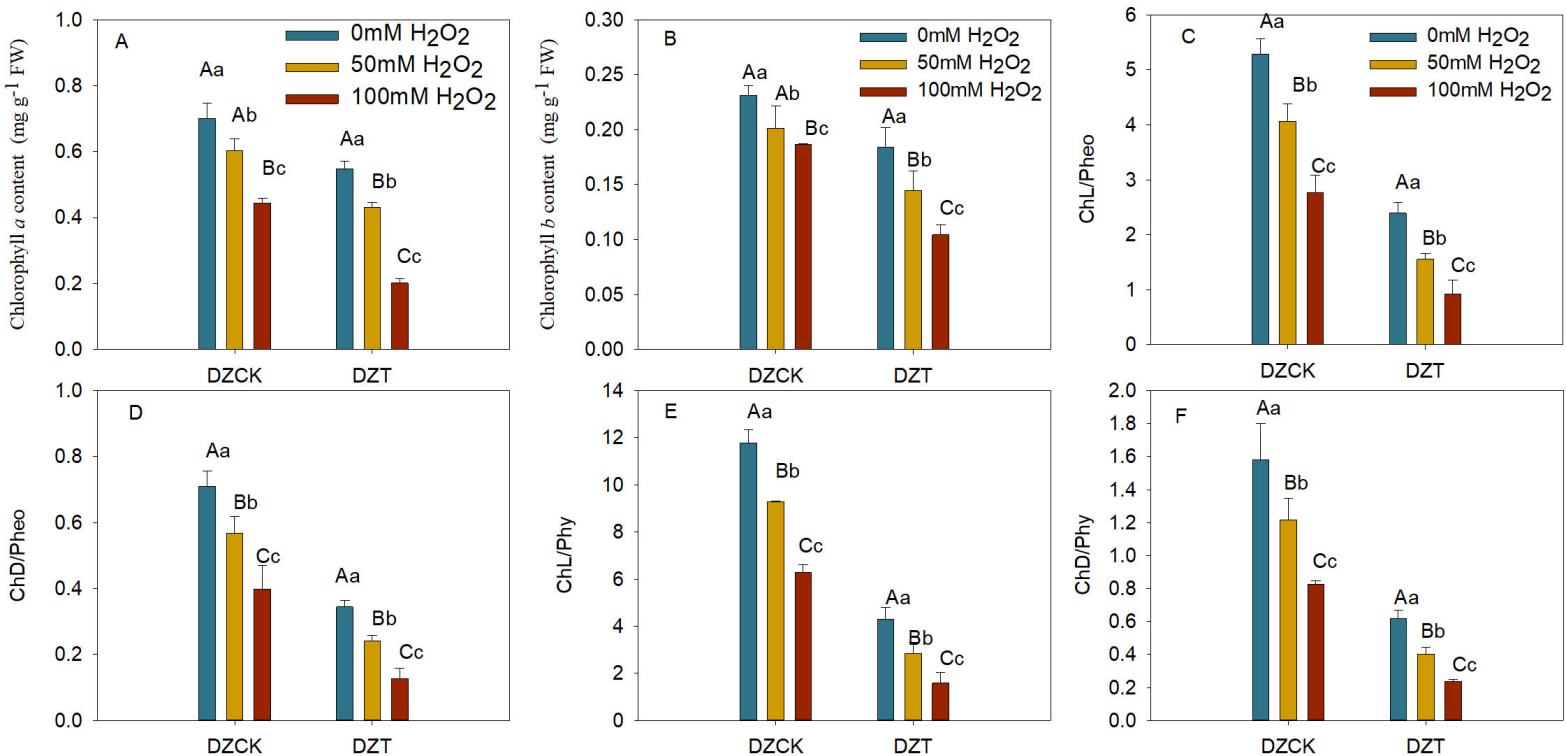
Assessing the Impact of Hydrogen Peroxide Treatment on Chlorophyll Metabolism *Ziziphus jujuba* Leaves Affected by Apolygus lucorum. **(A)** Chlorophyll a content in various jujube leaf samples. **(B)** Chlorophyll b content in different jujube leaf samples. **(C)** Ratio of Chlorophyll to Pheophorbide (Chl/pheo) in diverse jujube leaves. **(D)** Ratio of Chlorophyllide to Pheophorbide (ChD/pheo) in various jujube leaves. **(E)** Ratio of Chlorophyll to Pheophytin (Chl/phy) in different jujube leaves. **(F)** Ratio of Chlorophyllide to Pheophytin (ChD/phy) in diverse jujube leaves. DZCK, winter jujube without *A. lucorum;* DZT, winter jujube infested by *A. lucorum.* Data are presented as mean ± standard error (SE). Statistical significance was determined using *ANOVA*, with different lowercase letters indicating *p* < 0.05 and different uppercase letters indicating *p* < 0.01.

The application of exogenous H_2_O_2_ at 50 mM and 100 mM concentrations significantly impacted chlorophyll content in winter jujube leaves. In healthy leaves, the chlorophyll a content decreased by 11.16% (p = 0.038) and 30.97% (p < 0.01) for the 50 mM and 100 mM treatments, respectively, compared to the control group. In *A. lucorum*-infested leaves, the reduction was more pronounced, with decreases of 28.52% (p < 0.01) and 54.66% (p < 0.01) for the same treatments (**Figure 8A**).

Similarly, chlorophyll *b* content in healthy winter jujube leaves showed reductions of 13.05% (p = 0.021) and 19.39% (p < 0.01) for the 50 mM and 100 mM treatments, while in infested leaves, the decreases were 28.15% (p < 0.01) and 44.14% (p < 0.01) compared to the control (**Figure 8B**).

H_2_O_2_ treatment also significantly decreased the ratios of chlorophyll to pheophorbide and chlorophyllide to pheophorbide in winter jujube leaves. In *A. lucorum*-infested leaves, the chlorophyll/pheophorbide ratio decreased by 61.76% (p < 0.01) and 66.87% (p < 0.01) for 50 mM and 100 mM treatments, respectively (**Figure 8C**). The chlorophyllide/pheophorbide ratio decreased by 57.55% (p < 0.01) and 67.91% (p < 0.01) for the same treatments (**Figure 8D**).

The chlorophyll to pheophytin ratio in infested winter jujube leaves decreased by 69.45% (p < 0.01) and 74.86% (p < 0.01) under 50 mM and 100 mM H_2_O_2_ treatments (**Figure 8**), while the chlorophyllide/pheophytin ratio showed reductions of 66.95% (p < 0.01) and 71.37% (p < 0.01) for the same treatments (**Figure 8F**).

## 4 Discussion

### 4.1 Correlation between chlorophyll content reduction and photosynthetic efficiency decline after *A. lucorum* infestation.

Photosynthesis is a fundamental physiological process that underpins plant growth, development, and productivity by converting light energy into chemical energy to fuel metabolic activities (Briggs et al., 1985). However, this critical process is particularly vulnerable to biotic stress, such as pest infestations, which disrupt the photosynthetic machinery and lead to significant physiological damage (Bolton, 2009; Kangasjärvi et al., 2012). In this study, a pronounced reduction in photosynthetic efficiency was observed in winter jujube leaves following *A. lucorum* infestation (**Figure 2**). This decline is in line with previous research, which has demonstrated that biotic stressors such as pests and pathogens can adversely affect photosynthesis. For instance, *Phytophthora infestans* infection in tobacco (Bilgin et al., 2010) and *Diuraphis noxia* infestation in barley (Miller et al., 1994; Botha et al., 2006) both led to suppressed photosynthesis, underscoring the vulnerability of the photosynthetic process under pest attack. In addition to clarifying the impact of *A. lucorum* on jujube leaf photosynthesis, it is essential to consider strategies for mitigating this effect. Future research should explore these strategies to improve the resilience of jujube trees to *A. lucorum* infestation.

Chlorophyll, the primary pigment responsible for light absorption in photosynthesis, plays a pivotal role in determining photosynthetic efficiency (Senge et al., 2014). A reduction in chlorophyll content is directly correlated with a decrease in the plant’s capacity to capture light and convert it into energy. In this study, winter jujube leaves exhibited a substantial decline in both chlorophyll a and chlorophyll b content post-infestation (**Figure 1A**). This observation aligns with findings by Funayama-Noguchi and Terashima (2006) and Zanini et al. (2021), who reported a similar negative correlation between pest pressure and chlorophyll content in various plants. Moreover, our results indicate that the reduction in chlorophyll content was strongly correlated with the decline in net photosynthesis rate (Pn), especially in winter jujube (**Tables 1**, **2**), which further supports the well-established photosynthesis theory suggesting that decreased chlorophyll availability limits the plant’s ability to efficiently convert light energy into chemical energy (Farquhar et al., 1980; Björkman and Demmig, 1987).

In addition to chlorophyll, carotenoids, which serve a dual role in light absorption and photoprotection, also exhibited a decrease in content following *A. lucorum* infestation (**Figure 1**). Despite their critical role in preventing damage to the photosynthetic apparatus from excess light energy (Demmig-Adams and Adams, 1996), the reduction in carotenoid content was not significantly correlated with the decline in Pn (**Table 1**). This lack of correlation suggests that under the conditions of *A. lucorum* infestation, the reduction in chlorophyll content plays a more dominant role in determining photosynthetic efficiency than carotenoid levels. The findings imply that although carotenoids contribute to maintaining photosynthetic integrity, it is the loss of chlorophyll that primarily drives the observed decline in photosynthetic performance in winter jujube leaves post-infestation.

Collectively, these results emphasize the profound impact of *A. lucorum* on the photosynthetic machinery of winter jujube leaves, with chlorophyll degradation playing a central role in mediating the decline in photosynthetic efficiency. This study contributes to the understanding of how pest-induced stress can impair key physiological processes in plants, offering insights that could inform strategies to mitigate the impact of pest infestations on crop productivity.

### 4.2 Impairment of chlorophyll biosynthesis pathways in winter jujube under *A. lucorum* stress

The infestation by *A. lucorum* in winter jujube leaves significantly disrupts chlorophyll metabolism, which is intricately linked to the plant’s overall photosynthetic performance. Chlorophyll metabolism, comprising both synthesis and degradation, is fundamental to maintaining the integrity of the photosynthetic apparatus. The data from this study suggest that the balance between these two processes is severely skewed during infestation, leading to critical declines in photosynthetic efficiency.

Chlorophyll biosynthesis, essential for maintaining the plant’s capacity for light absorption and energy conversion, was significantly impaired following *A. lucorum* infestation. The downregulation of chlorophyll biosynthesis genes, such as HEMA and HEMB, likely contributes to a metabolic bottleneck that limits the plant’s ability to produce chlorophyll under stress. These findings are consistent with previous studies that have shown reduced chlorophyll synthesis under biotic stress conditions (Nabity et al., 2009; Zvereva et al., 2010). These genes play crucial roles in converting glutamate to ALA and then into porphyrins, the core precursors for chlorophyll (**Figure 3**). The downregulation of these genes by 20–30% reflects a metabolic bottleneck that limits the plant’s ability to maintain adequate chlorophyll levels under stress.

This disruption extends to later stages of chlorophyll synthesis, as indicated by reduced expression of *chlG* and *CAO*, enzymes responsible for converting chlorophyllide to chlorophyll a and chlorophyll *b*, respectively. These reductions directly contribute to the observed decline in chlorophyll content (**Figure 1A**).

### 4.3 Acceleration of chlorophyll degradation pathways in response to *A. lucorum* infestation

In parallel with the suppression of chlorophyll biosynthesis, *A. lucorum* infestation triggered an upregulation of chlorophyll degradation pathways, suggesting that the plant is actively accelerating the breakdown of chlorophyll molecules. This response likely reflects a physiological adaptation aimed at reducing photodamage and mitigating oxidative stress under pest-induced stress.

The upregulation of *PAO* and *RCCR*, by 39.15% and 61.37%, respectively (**Figure 3**), suggests an increase in the catabolism of chlorophyll molecules into pheophytin a and pheophorbide (**Figure 4C**). These breakdown products are critical intermediates in the chlorophyll degradation pathway, and their accumulation indicates that the plant is shifting towards chlorophyll catabolism to manage the excess energy and ROS that could be generated under stress. The chlorophyll/pheophytin and chlorophyllide/pheophytin ratios show dramatic reductions of over 60% (**Table 2**), further supporting the enhanced degradation over synthesis in this process. This was in line with the findings that stress conditions could activate chlorophyllase, the enzyme responsible for the initial step in chlorophyll degradation (Jimenez et al., 1995; McAuslane et al., 2004).

Chlorophyll degradation, while protective in reducing ROS production, comes with significant costs. As chlorophyll levels drop, the plant’s ability to sustain photosystem activity declines, leading to reduced energy output. This is particularly critical during periods of biotic stress, where energy demands are higher due to the need for defense mechanisms, such as the synthesis of defensive compounds or the activation of pathogen resistance genes. The negative correlation between Pn and pheophorbide content (Pearson correlation coefficient = -0.556, p = 0.011) reflects this trade-off: the plant reduces photodamage by degrading chlorophyll but at the cost of reduced photosynthetic performance.

Interestingly, the Gene Ontology (GO) analysis supports this physiological shift, with significant changes in chloroplast-associated pathways, including those involved in chlorophyll biosynthesis and photosynthesis (**Figure 2**). The downregulation of transcription factors related to chlorophyll synthesis, combined with the upregulation of those associated with chlorophyll degradation, suggests that the plant undergoes a broad reprogramming of its metabolic priorities. This reflects a common stress response in which the plant sacrifices photosynthetic efficiency to protect itself from further damage (Hörtensteiner, 2009).

### 4.4 Chlorophyll depletion impaired photosystem activity and triggered oxidative stress in winter jujube under A. lucorum infestation

The reduction in chlorophyll content due to A. lucorum infestation significantly impairs the photosynthetic machinery and electron transport chain (ETC) in winter jujube. This aligns with established plant responses to biotic stress, where chlorophyll depletion leads to photosynthetic damage and the disruption of electron flow in Photosystem II (PSII). The Photochemical Performance Index (PI_ABS_), a sensitive indicator of photosynthetic efficiency, exhibited a significant decrease post-infestation, suggesting that winter jujube is particularly vulnerable to photosynthetic damage (Bussotti et al., 2007b).

The marked reduction in Fv/Fm and Fv/Fo ratios further supports the idea that PSII efficiency is diminished in winter jujube (**Table 3**), indicating that less energy is being converted to photochemical reactions. This decrease in electron transfer efficiency (ψ(Eo)) (**Figure 6**) suggests that the electron transport chain is compromised, leading to electron accumulation and potential ROS generation, particularly superoxide anions (O_2_^-^) and H_2_O_2_ (Edreva, 2005; Pospíšil, 2016). The reduced ψ(Eo) implies that the plant’s ability to efficiently transfer electrons within the photosynthetic apparatus is severely impaired, thus limiting overall photosynthetic performance.

The observed increase in δ(Ro) (**Figure 6**), representing non-photochemical energy dissipation, further underscores this shift toward energy loss as heat rather than productive energy conversion. The heightened δ(Ro) suggests a response to mitigate photodamage, but this also increases the risk of ROS generation due to an imbalance in energy utilization (Andrews et al., 1995; Kalaji et al., 2014). This is consistent with the substantial increase in O_2_^-^ and H_2_O_2_ levels observed in winter jujube leaves (**Figure 7A**). The overproduction of ROS in chloroplasts exacerbates oxidative stress, further damaging the photosynthetic apparatus and reducing the plant’s ability to recover from infestation.

The increase in the Absorption Flux per Reaction Center (ABS/RC) suggests an adaptation by winter jujube to capture more light energy per reaction center (**Figure 6**), potentially as a response to diminished chlorophyll levels (Wang et al., 2022). However, this adaptation does not correspond to improved energy conversion efficiency. Instead, the increase in ABS/RC reflects the plant’s attempt to compensate for the loss of functional chlorophyll, but it ultimately leads to an increase in dissipated energy (DIo/RC), as evidenced by the 95.69% rise in this parameter post-infestation (**Figure 6**). This indicates that more energy is being lost as heat rather than being channeled into productive photosynthetic processes, further reducing photosynthetic efficiency (Oukarroum et al., 2015).

The significant decrease in Electron Transport per Reaction Center (ETo/RC) (**Figure 6**) further demonstrates the inability of winter jujube to maintain efficient electron transport within the photosystems. The disruption of ETC is accompanied by a decline in Reduction of Electron Transport per Reaction Center (REo/RC), which directly impacts the production of NADPH and the plant’s ability to fuel photosynthetic reactions. These declines collectively signal a severely compromised photosynthetic apparatus in winter jujube.

The decline in photosynthetic efficiency and the disruption of the ETC trigger the accumulation of ROS, which contribute to oxidative stress in winter jujube leaves. The significant increases in superoxide anion (O_2_^-^) and H_2_O_2_ content (**Figure 7A**) indicate that ROS are overproduced due to the impaired electron transport chain. The increase in ROS, including superoxide anions and hydrogen peroxide, likely leads to lipid peroxidation in chloroplast membranes, further compromising the photosynthetic machinery and exacerbating the damage to the plant’s cellular structure.

The plant’s response to oxidative stress is reflected in the upregulation of antioxidant enzymes, including superoxide dismutase (SOD), ascorbate peroxidase (APX), and catalase (CAT) (**Figures 7D-G**). Although the activation of these enzymes helps mitigate some of the damage by detoxifying ROS, their increased activity indicates the severity of oxidative stress (Ort et al., 2010; Zhu et al., 2010; Gu et al., 2017). However, despite this antioxidant response, the damage inflicted by ROS on the photosynthetic apparatus—particularly the loss of chlorophyll and impaired electron transport—limits the plant’s ability to sustain photosynthesis under stress conditions (Yoo et al., 2003; Bussotti et al., 2007a; Wang et al., 2015). Similar findings have been reported in studies on aphid-infested crops, where pest-induced stress caused significant declines in chlorophyll content and photosynthetic efficiency (Guo et al., 2018). These comparisons underscore the generality of pest-induced oxidative stress in plants.

### 4.5 The role of H_2_O_2_ in accelerating chlorophyll degradation and compromising photosynthetic function in winter jujube leaves

The application of exogenous H_2_O_2_ significantly intensified chlorophyll degradation in *A. lucorum*-infested winter jujube leaves, underlining the critical role of oxidative stress in exacerbating damage to photosynthetic machinery. This study reveals how oxidative stress—both from internal sources during pest infestation and from external application—drives accelerated chlorophyll catabolism, leading to a dramatic decline in photosynthetic capacity. This heightened vulnerability to H_2_O_2_ treatments in infested leaves underscores the complex interaction between biotic and abiotic stress factors.

In healthy winter jujube leaves, H_2_O_2_ treatment alone led to moderate reductions in chlorophyll a and chlorophyll b content. However, in *A. lucorum*-infested leaves, these reductions were magnified, with chlorophyll *a* levels decreasing by over 54% and chlorophyll *b* by 44% under 100 mM H_2_O_2_ treatment (**Figures 8A, B**). This sharp contrast highlights the synergistic effect of biotic stress and oxidative stress, where the pest infestation primes the chloroplasts for further degradation, and exogenous H_2_O_2_ acts as a catalyst to accelerate these processes (Bhattacharjee, 2019).

The pronounced chlorophyll loss in infested leaves likely results from the combined ROS generated during pest infestation and the additional oxidative load from H_2_O_2_. The increased oxidative burden overwhelms the plant’s antioxidant defenses, promoting chlorophyll breakdown. This interplay emphasizes that *A. lucorum* not only damages chloroplasts through direct feeding but also indirectly weakens the plant’s resilience to other stressors by promoting oxidative imbalances.

The significant reductions in chlorophyll/pheophorbide and chlorophyllide/pheophorbide ratios post-H_2_O_2_ treatment indicate a marked shift in the chlorophyll degradation pathway. The increased presence of pheophorbide, a key degradation product, suggests that chlorophyll catabolism is significantly upregulated in response to oxidative stress. In infested leaves, the chlorophyll to pheophorbide ratio decreased by up to 66.87% (**Figure 8C**), showing that chlorophyll degradation was not only initiated but accelerated under stress conditions.

Additionally, the decline in chlorophyll to pheophytin ratios by nearly 75% points to the increased activity of Mg-dechelation enzymes, responsible for converting chlorophyll into pheophytin (**Figure 8D**). This rapid conversion suggests that H_2_O_2_ treatment intensifies the breakdown of chlorophyll into its degradation products, hastening the plant’s inability to maintain functional light-harvesting complexes.

These findings illustrate the heightened sensitivity of the chlorophyll degradation pathway to oxidative stress, particularly in leaves already compromised by *A. lucorum.* The substantial reduction in these critical pigment ratios reveals how pest-induced oxidative stress primes chlorophyll for faster degradation, and how H_2_O_2_ further accelerates this process by promoting the breakdown into pheophytin and pheophorbide.

## 5 Conclusion

This study demonstrates the significant impact of *A. lucorum* infestation on chlorophyll metabolism and photosynthetic performance in winter jujube leaves. The decline in chlorophyll content was closely associated with reduced photosynthetic efficiency, evidenced by decreases in key parameters such as PI_ABS_ and ψ(Eo). The infestation disrupted chlorophyll biosynthesis by downregulating critical genes like *HEMA*, *HEMB*, *HEMC*, *chlG*, and *CAO*, while simultaneously accelerating chlorophyll degradation through upregulation of genes such as *PAO* and *RCCR*. The production of ROS triggered by the infestation exacerbated chlorophyll breakdown, further damaging the photosynthetic machinery. Exogenous H_2_O_2_ intensified this degradation, particularly in infested leaves, underscoring the combined effects of biotic and abiotic stress on chlorophyll loss. Despite an upregulation of antioxidant enzymes, the plant’s ability to maintain photosynthesis remained compromised. These findings highlight the need for strategies to mitigate oxidative stress and protect chlorophyll content to preserve crop productivity under pest pressure. Future research should focus on developing strategies to mitigate the effects of *Apolygus lucorum* infestation, including the use of antioxidant treatments or pest control methods to reduce the impact on photosynthesis.

## Data Availability

The original contributions presented in the study are publicly available. This data can be found here: NCBI SRA, accession SRR32076166 and SRR32076165.
